# OsWRKY28 Regulates Phosphate and Arsenate Accumulation, Root System Architecture and Fertility in Rice

**DOI:** 10.3389/fpls.2018.01330

**Published:** 2018-09-12

**Authors:** Peitong Wang, Xuan Xu, Zhong Tang, Wenwen Zhang, Xin-Yuan Huang, Fang-Jie Zhao

**Affiliations:** State Key Laboratory of Crop Genetics and Germplasm Enhancement, College of Resources and Environmental Sciences, Nanjing Agricultural University, Nanjing, China

**Keywords:** OsWRKY28, rice, phosphate, arsenate, root, fertility

## Abstract

WRKYs are transcriptional factors involved in stress tolerance and development of plants. In the present study, we characterized *OsWRKY28*, a group IIa WRKY gene, in rice, because its expression was found to be upregulated by arsenate exposure in previous transcriptomic studies. Subcellular localization using YFP–OsWRKY28 fusion protein showed that the protein was localized in the nuclei. Transgenic rice plants expressing *pOsWRKY28*::GUS suggested that the gene was expressed in various tissues in the whole plant, with a strong expression in the root tips, lateral roots and reproductive organs. The expression of *OsWRKY28* was markedly induced by arsenate and other oxidative stresses. In a hydroponic experiment, loss-of-function mutation in *OsWRKY28* resulted in lower accumulation of arsenate and phosphate concentration in the shoots. The mutants showed altered root system architecture, with fewer lateral roots and shorter total root length than wild-type plants. In a soil pot experiment, the mutants produced lower grain yield than wild-type because of reduced fertility and smaller effective tiller numbers. Transcriptomic profiling using RNA-seq showed altered expression in the mutant of genes involved in the biosynthesis of phytohormones, especially jasmonic acid (JA). Exogenous JA treatments mimicked the phenotypes of the *oswrky28* mutants with inhibited root elongation and decreased arsenate/phosphate translocation. Our results suggested that *OsWRKY28* affected arsenate/phosphate accumulation, root development at the seedling stage and fertility at the reproductive stage possibly by influencing homeostasis of JA or other phytohormones.

## Introduction

Inorganic arsenic (As) is a non-threshold carcinogen. Humans are exposed to inorganic As mainly through drinking water and food. Arsenic contamination in paddy soils is a common problem worldwide due to mining and smelting, irrigation of As-laden groundwater, and uses of As-containing agrochemicals. Contaminated paddy soils can result in elevated accumulation of As in rice grain and pose a significant risk to the health of people consuming rice as their staple food (Meharg, 2004; Zhao et al., 2010; Banerjee et al., 2013). At high levels of contamination, As can cause phytotoxicity and substantial yield losses (Panaullah et al., 2009; Khan et al., 2010; Huhmann et al., 2017). It is therefore important to understand how rice plants take up As and respond to As toxicity stress.

In flooded paddy soils, arsenite [As(III)] is the predominant chemical species of As, although arsenate [As(V)] can still account for 5 to 20% of the total soluble As in the soil solution (Khan et al., 2010; Stroud et al., 2011). As(III) is taken up by rice roots via NIP aquaporin channels, such as OsNIP2;1 (OsLsi1) and OsNIP3;2, and the silicon transporter OsLsi2 (Ma et al., 2008; Chen et al., 2017). In contrast, As(V) is a chemical analog of phosphate (Pi) and is taken up by Pi transporters. There are 13 genes in the Pht1 family (*OsPT1*–*OsPT13*) encoding Pi transporters in the rice genome (Paszkowski et al., 2002). The roles of *OsPT1, OsPT2, OsPT4, OsPT6, OsPT8, OsPT9* and *OsPT10* in Pi uptake, translocation and homeostasis have been investigated (Ai et al., 2009; Jia et al., 2011; Sun et al., 2012; Wang et al., 2014; Zhang et al., 2015). Among the *OsPT* genes investigated, *OsPT1, OsPT4*, and *OsPT8* are known to be involved in either As(V) uptake or translocation (Kamiya et al., 2013; Wang et al., 2016; Cao et al., 2017; Ye et al., 2017). A commonly reported mechanism of As(V) tolerance is the down-regulation of the high affinity Pi transport systems, thus restricting As(V) uptake to adapt to high As soils (Meharg and Hartley-Whitaker, 2002). When *Arabidopsis thaliana* plants were exposed to As(V), the expression of the transcriptional factor gene *AtWRKY6* was induced rapidly by As(V) exposure, which acts to depress the expression of Pi/As(V) transporter genes and decrease As(V) uptake (Castrillo et al., 2013). This mechanism was thought to be a strategy for *A. thaliana* to adapt to As in the environment during evolution.

WRKY (containing the WRKY protein domain) is one of the largest families of transcription factors, which is classified based on the sequence of 60 amino acid residues in WRKY proteins, including N-terminal WRKYGQK continuous heptapeptide and C-terminal metal chelated zinc finger structure (Eulgem et al., 2000; Rushton et al., 2010). WRKY transcriptional factors play important roles in the regulation of plant growth, development and apoptosis, and responses to biotic and abiotic stresses (Ulker and Somssich, 2004; Eulgem and Somssich, 2007; Ross et al., 2007; Pandey and Somssich, 2009; Song et al., 2010; Agarwal et al., 2011; Chen et al., 2012). There are 74 and 109 WRKY members in the *Arabidopsis* and rice genome, respectively (Ross et al., 2007; Rushton et al., 2010). It is not known if WRKY transcription factors are involved in regulating As(V) uptake or translocation in rice, as has been shown in *A. thaliana* with AtWRKY6 (Castrillo et al., 2013). A previous transcriptomic study showed that some *OsWRKY* genes, especially *OsWRKY28*, were highly responsive to As(V) (Chakrabarty et al., 2009), implying a possible role of OsWRKY28 in regulating As(V) uptake or tolerance in rice.

*OsWRKY28* belongs to the group IIa of WRKY genes that also include *OsWRKY62, OsWRKY71* and *OsWRKY76* in rice (Eulgem et al., 2000; Peng et al., 2008). *OsWRKY28* was previously reported to play a negative role in the defense against blast fungus in rice (Peng et al., 2010; Delteil et al., 2012; Chujo et al., 2013). In the present study, we investigated the role of *OsWRKY28* in regulating As(V) accumulation and plant growth in rice. We found that the gene was greatly induced by the exposure to As(V) and other heavy metals or oxidative stress. Mutation in *OsWRKY28* decreased the accumulation of As and Pi in the shoots but did not affect the expression of Pi transporter genes. In addition, root growth and fertility were also altered in *oswrky28* mutants. We further investigated the possible involvement of the plant hormone, jasmonic acid (JA), in the regulation of As(V) uptake by OsWRKY28.

## Materials and Methods

### Plant Materials and Growth Conditions

Two T-DNA insertion mutants of *OsWRKY28* were obtained in the present study, *oswrky28-1* (PFG_1B-20614.L from Postech in the Dongjin (DJ) background) and *oswrky28-2* [RMD_04Z11OD34 from Huazhong Agricultural University in the Zhonghua 11 (ZH11) background]. Both WT cultivars belong to the *Japonica* rice (*Oryza sativa* L. subsp. *japonica*). Seeds of WT and mutants were surface-sterilized and germinated on 1/2 MS (Murashige and Skoog) for 7 days. Seedlings were transferred to half-strength Kimura B solution for 3 weeks(Yan et al., 2016). The compositions of the nutrient solution were (in μM) 91 KH_2_PO_4_, 270 MgSO_4_, 180 (NH_4_)_2_SO_4_, 90 KNO_3_, 180 Ca (NO_3_)_2_, 3 H_3_BO_3_, 0.5 MnCl_2_, 1 (NH_4_)_6_Mo_7_O_24_, 0.4 ZnSO_4_, 0.2 CuSO_4_, 20 NaFe(III)-EDTA and pH was adjusted to 5.6. The solution was renewed every 3 days. After 3 weeks, plants were treated with 5 μM As(V) in a fresh half-strength Kimura B solution for 3 days before analysis of As and Pi concentrations in the roots and shoots.

### Generation of *OsWRKY28* Overexpression Lines and *pOsWRKY28*-GUS Lines

To generate *OsWRKY28* overexpression lines, a 1,191-bp open reading frame of *OsWRKY28* from cv Nipponbare was amplified using the primers listed in **Supplementary Table S1**. The fragments were cloned into pTCk303 vector (Wang et al., 2004). Agrobacterium-mediated transformation was performed, and T1 plants were selected and used for phenotyping.

To determine the expression pattern of *OsWRKY28* in different rice tissues, a 2,059-bp promoter sequence of *OsWRKY28* was amplified and inserted into the binary vector pS1aGUS-3 (Zhang et al., 2015) and transformed into rice (Nipponbare) using the Agrobacterium-mediated transformation method. *Agrobacterium tumefaciens* strain EHA105 was used in the transformation. The primers used are listed in **Supplementary Table S1**.

### Tissue and Subcellular Localization Analysis

The histochemical analysis of GUS (β-glucuronidase) activity of samples was incubated in GUS reaction mixture at 37°C overnight. Green tissues were bleached by ethanol prior to observation. The stained tissues were photographed by using an OLYMPUS SZX7 stereo microscope. To identify the subcellular localization of OsWRKY28, the full-length cDNA of *OsWRKY28* was amplified and inserted into the vector pEarleyGate 101 (Gao et al., 2017) containing the coding sequence for the yellow fluorescent protein (YFP). The vector of 35S:OsWRKY28-YFP was transiently expressed in tobacco (*Nicotiana benthamiana*) leaves by Agrobacterium-mediated infiltration. The fluorescence of tobacco leaves was imaged 3 days after infiltration using a confocal laser scanning microscope (LSM410; Carl Zeiss). The excitation wavelength for eYFP fluorescence was 488 nm, and fluorescence was detected at 500–542 nm. The nuclear dye 4′,6-diamidino-2-phenylindole (DAPI) was used to stain the nucleus. The primers used for tissue and subcellular localization analysis are listed in **Supplementary Table S1**.

### Gene Expression Analysis

The response of *OsWRKY28* expression to the exposure to 1.5 μM As(V), 20 μM As(III), 3 μM Cd, 2 μM Cu or 500 μM H_2_O_2_ was tested in 3-day old rice seedlings (cv Nipponbare). After exposure for 12 h, roots were collected for the extraction of total RNA. The effect of *OsWRKY28* mutation on the expression of Pi transporter genes was also determined by comparing the transcript abundances of Pi genes in *oswrky28-1* and *oswrky28-2* mutants and their WT plants grown hydroponically under normal conditions for 4 weeks. To analyze the expression of phosphate transporter genes under P-sufficient and P-deficient conditions, plants were pre-cultivated in the above half-strength Kimura B solution for 2 weeks and then divided into two groups supplied with (91 μM) or without Pi for 1 week. KCl was added to P-deficient group to maintain the same K concentration. Roots and shoots were collected for quantification of the expression of *OsIPS1, OsPT1, OsPT2, OsPT6* and *OsPT8*. Total RNA was extracted from roots using a RNA Extraction Kit (BioTeke, Beijing, China) according to the manufacturer’s instructions. The genomic DNA was removed and the total RNA was converted to cDNA using the HiScript II 1st Strand cDNA Synthesis Kit (Vazyme). Quantitative real-time PCR was performed on a BioRad CFX96 real time system and the products were labeled using the SYBR Green master mix (Vazyme). *OsHistone3* and *OsACTIN* were used as the reference genes. The primers used for real-time RT-PCR analysis of Pi transporter and As related genes are listed in **Supplementary Table S1**.

### RNA-seq and Data Analysis

Root and shoot transcriptomes in 4-week old plants of *oswrky28-1* and WT (Dongjin) grown under normal hydroponic conditions were analyzed by RNA-seq. Total RNA was extracted as described above. Truseq^TM^ RNA sample prep Kit (Illumina) was used to construct libraries. Double-stranded cDNA was sequenced as 150bp^∗^2 using the Illumina GA Genome Analyzer paired-end pipeline. The original data set has been deposited in the NCBI SRA database (access no. SRP131249). The expression levels were calculated as read counts derived from FPKM (Fragments Per Kilobase of exon model per Million mapped reads) based on the number of uniquely positioned reads overlapping the exon regions. The threshold of *P*-values was determined by FDR in multiple tests. In this study, FDR ≤ 0.05 and the gene expression fold change ≥ 2 were used as the threshold to identify genes that showed significantly different expression between the mutant and WT.

### Soil Pot Experiment

A soil pot experiment was performed with four replications in a greenhouse using the soil collected from the experimental farm of Nanjing Agricultural University. The soil contains 35 mg kg^-1^(1.13 mmol kg^-1^) available Pi (the Olsen P method) and 12 mg kg^-1^(0.16mmol kg^-1^) total As (McGrath and Cunliffe, 1985) and has a pH value of 6.4. Twelve kg air-dried soil was placed into a 15 L plastic pot. Phosphate fertilizer (KH_2_PO_4_) and sodium arsenate were mixed into the soil to add 100 mg kg^-1^ Pi and 5 mg kg^-1^ As. Two-week old seedlings of *oswrky28-1, oswrky28-2* and their WTs were transplanted into each pot (one plant for each line per pot). Plants were grown under flooded conditions in a net enclosure with natural sunlight and ambient temperature during summer season of 2015. Plants were harvested at grain maturity.

### Determination of Total As, Total P and Pi Concentrations

Rice plants from hydroponic experiments were separated into roots and shoots. Plants from the soil pot experiment were separated into straw and grain. Plant samples were dried at 60°C for 2 days and ground to fine powder. Dry plant samples were digested with HNO_3_:HClO_4_ (85:15, v/v) following the method described by Zhao et al. (1994). Total As, P and other element concentrations were determined by inductively coupled plasma mass spectrometry (ICP-MS, Perkin Elmer NexION-300x). To determine Pi concentration, fresh plant samples were collected, extracted with 10% (w/v) of perchloric acid and quantified using a molybdenum blue method (Zhou et al., 2008).

### Measurements of Root Length, Total Surface Area, and Numbers of Tips

Plants of *oswrky28-1, oswrky28-2* and their WTs were grown hydroponically under normal conditions for 4 weeks. The images of the roots of individual plants were acquired using a scanner (LA1600+ scanner, Canada), and the root-related parameters (total length, total surface area, and numbers of tips) were analyzed using Win-rhizo software (Win-rhizo 2003b, Canada). Data were recorded from four individual plants from each line per treatment.

### Data Analysis

All data were analyzed by ANOVA, followed by comparisons of means using the Tukey’s test (*P* < 0.05). Statistical analyses were performed using SPSS 20.

## Results

### *OsWRKY28* Is Highly Responsive to Arsenate and Other Abiotic Stresses

**FIGURE 1:**
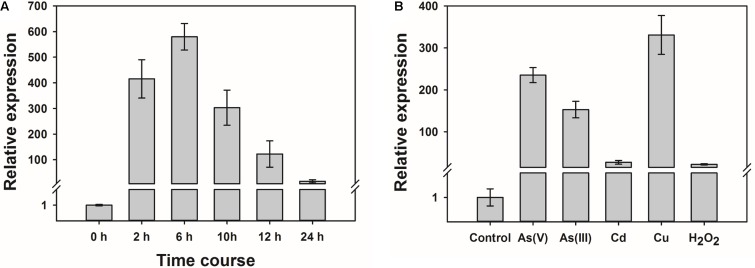
The expression of *OsWRKY28* was responsive to arsenate and other oxidative stress. **(A)** Time course of the expression level of *OsWRKY28* in roots. The plants were exposed to 1.5 μM As(V) without Pi supply for 24 h. **(B)** The transcript levels of *OsWRKY28* exposed to other oxidative stresses for 12 h. The plants were exposed to 1.5 μM As^5+^, 20 μM As^3+^, 3 μM Cd, 2 μM Cu, 500 μM H_2_O_2_ without Pi supply. The relative expression of *OsWRKY28* were normalized to the expression level at 0 h **(A)** or without treatment, respectively. OsHistone3 was used as the reference gene. Data are means ± SE (*n* = 3 biological replicates).

Quantitative RT-PCR was used to determine the level of expression of *OsWRKY28*. The expression of *OsWRKY28* increased rapidly in response to the exposure to 1.5 μM As(V) (**Figure 1A**), which was found to be the effect concentration of As(V) resulting in a 50% inhibition (EC_50_) in root growth for cv Nipponbare (in the absence of Pi in the nutrient solution) (Wang et al., 2016). The transcript abundance increased by 400 and 600 fold after As(V) exposure for 2 and 6 h, respectively. After 6 h, the expression of *OsWRKY28* started to decrease, reaching a level at 24 h that was approximately 10 times the initial level (**Figure 1A**).

Several *WRKY* genes have been reported to be responsive to abiotic stresses. We therefore tested if *OsWRKY28* also responds to other abiotic stresses. Three-day old plants (cv Nipponbare) were exposed to 1.5 μM As(V), 20 μM As(III), 3 μM Cd, 2 μM Cu or 500 μM H_2_O_2_ for 12 h. The concentrations of As(V), As(III) and Cd used were approximately equivalent to their respective EC_50_ for root growth inhibition, whereas the concentration of Cu used caused total inhibition of root growth. The concentration of H_2_O_2_ used was the same as that used by Liu et al. (2014). The expression of *OsWRKY28* was responsive to all stresses tested, with 200, 150, 20, 300, and 20 fold increase in the transcript level in response to As(V), As(III), Cd, Cu and H_2_O_2_, respectively (**Figure 1B**). The experiment was repeated with 3-week-old plants and similar responses were obtained (**Supplementary Figure S1**).

### The Subcellular Localization OsWRKY28 and the Tissue Expression Pattern of *OsWRKY28*

**FIGURE 2:**
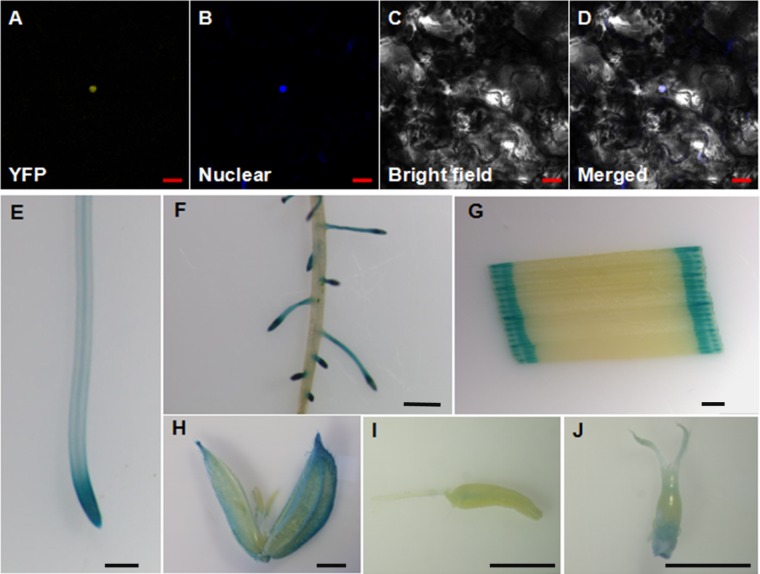
Subcellular localization and expression pattern of *OsWRKY28*. **(A–D)** Subcellular localization and expression pattern of *OsWRKY28*. Confocal image of tobacco (*Nicotiana benthamiana*) epidermal cells expressing the OsWRKY28:eYFP fusion protein **(A)**. **(B)** Confocal images of epidermal cells stained by a nuclear specific dye 4,6-diamidino-2-phenylindole (DAPI). **(C)** Bright-field images. **(D)** The merged image of **(A–C)**. Bar = 20 μm. **(E–J)** Expression pattern of *OsWRKY28* was revealed by GUS-staining. Primary root **(E)**, lateral root, **(F)**, leaf **(G)**, caryopsis at the flower development stage **(H)**, stamen **(I)**, stigma and ovary **(J)**. Four independent GUS lines were observed, and the same results were obtained. Bar = 1 mm.

OsWRKY28 was reported to be localized to the nucleus when the gene was transiently expressed in onion epidermal cells (Chujo et al., 2013). To verify its subcellular localization, we constructed YFP-*OsWRKY28* fusion protein driven by the cauliflower mosaic virus 35S promoter and transfected the derived expression vector into tobacco (*Nicotiana benthamiana*) epidermal cells. The YFP-OsWRKY28 signal was observed in the same position as the nucleus dyed by DAPI (**Figures 2A–D**). The results indicate that the OsWRKY28 protein is localized to the nucleus.

To investigate the tissue expression pattern of *OsWRKY28* in rice, a 2,059-bp promoter region together with the first exon and the first intron of *OsWRKY28* was amplified and fused to the GUS reporter gene. Subsequently, the constructs were transformed into rice (cv Nipponbare). The transgenic plants showed GUS activities in the primary roots, lateral roots, leaves, caryopses, stamens, stigma and ovary (**Figures 2E–J**). The GUS activity was particularly strong in the root tips and the lateral roots. The results were consistent with the gene expression pattern from RiceXPro database (**Supplementary Figure S2**). These results indicate that *OsWRKY28* was expressed in the whole plant and at different growth periods of rice.

### *OsWRKY28* Mutation Affects Phosphate and Arsenic Concentrations in the Shoots

**FIGURE 3:**
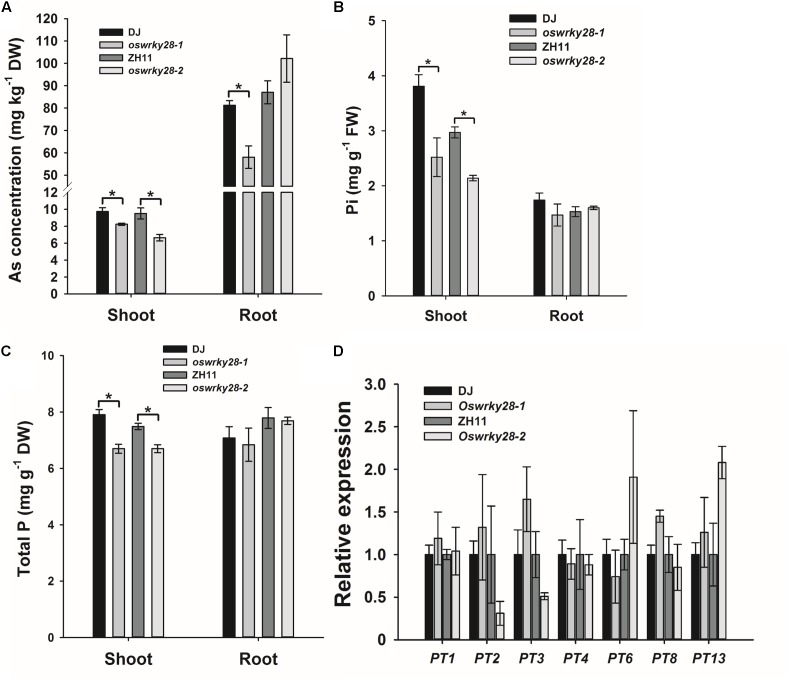
Arsenate and phosphate uptake and the expression levels of phosphate transporter genes in WT and *oswrky28* mutants. **(A)** Arsenic concentrations in shoots and roots of WT and *oswrky28*. **(B,C)** The concentrations of P **(B)**, and total phosphorus concentrations **(C)** in roots and shoots of WT and *oswrky28.* Plants were hydroponically cultivated in 1/2 kimura solution for 3 weeks and then exposed to 5 μM As(V) for 3 days. **(D)** The expression levels of different phosphate transporter genes in the roots of WT and *oswrky28* without arsenate treatment. Data are means ± SE (*n* = 4). FW, fresh weight, DW, dry weight. Asterisks indicate significantly different at *P* < 0.05 (Tukey’s test).

Because *OsWRKY28* was induced by As(V), we investigated if the gene regulates the uptake and/or distribution of As(V) and Pi. We obtained two T-DNA insertion mutants (*oswrky28-1* and *oswrky28-2*) in the DJ and ZH11 background, respectively (**Supplementary Figure S3**). T-DNA was inserted in the promoter region of *OsWRKY28* in both mutants, resulting in 96 and 94% decrease in the abundance of the gene transcript in *oswrky28-1* and *oswrky28-2*, respectively, compared with WTs (**Supplementary Figure S3C**). The two mutants and their respective WTs were grown in normal hydroponic culture with 100 μM Pi for 3 weeks, and then exposed to 5 μM As(V) for 3 days. ANOVA showed significant (*P* < 0.05) differences in the shoot concentrations of As and Pi between mutants and WTs, with the mutants accumulating 16–31% lower concentrations of As, 28–34% lower concentrations of Pi, and 11–16% lower concentrations of total P in the shoots than WT (**Figures 3A–C**). The *oswrky28-1*mutant also showed a significantly lower As concentration in the roots than its WT (DJ), but there was no significant difference between *oswrky28-2* and WT (**Figure 3A**). There were also no significant differences between mutants and WTs in the concentrations of Pi or total P in the roots (**Figures 3B,C**). The results suggest that *OsWRKY28* may play a role in regulating the translocation of As(V) and Pi from roots to shoots. In contrast, no consistent differences in the concentrations of Fe, Mn, Cu, or Zn in the shoots or roots were found between WT and *oswrky28* mutants (**Supplementary Figure S4**), suggesting that the decreased As and P concentrations in the mutants were specific to these elements.

*OsWRKY28* overexpression lines were also generated and compared with WT in a hydroponic experiment (**Supplementary Figure S5**). However, the root and shoot growth did not show consistent differences between the overexpression lines and control plants. P or As concentrations of the roots and shoots were similar in the overexpression lines and control plants (**Supplementary Figure S5**).

To investigate whether *OsWRKY28* regulates the expression of the genes involved in Pi uptake and translocation, quantitative RT-PCR was used to determine the expression levels of 12 Pi transporter genes in the OsPHT1 family and *OsPHO1;2* in the roots of mutants and WTs treated with As(V) for 3 d. *OsPT11* was not tested because of its specifically expression only upon symbiosis with arbuscular mycorrhiza in roots (Paszkowski et al., 2002; Yang et al., 2012). *OsPHO1;2* was included because of its function in the root-to-shoot translocation of Pi (Secco et al., 2010). Among the 13 genes examined, *OsPT1, OsPT2, OsPT3, OsPT4*, and *OsPT8* showed relatively high levels of transcript abundance, whereas *OsPT6, OsPT7, OsPT9, OsPT10*, and *OsPT13* were expressed at low levels, and *OsPT5* and *OsPT12* could not be detected (**Figure 3D**). There were no consistent differences in the expression of any of the 13 genes tested between mutants and WTs. To investigate further whether *OsWRKY28* was involved in the regulation of the expression of Pi transporter genes, we grew the mutants and wild-types at both P sufficient and deficient conditions. The transcript levels of *OsPT1, OsPT2, OsPT6, OsPT8*, and *OsIPS1*, a phosphate starvation maker gene, were quantified. As expected, the expression of *OsIPS1* in both roots and shoots was greatly increased under P deficient conditions (**Supplementary Figure S6**). The expression of *OsPT2* in the roots and *OsPT6* in both roots and shoots was also increased by P deficiency. However, there were no consistent differences between mutants and their wild-types in the expression levels of these Pi transporter genes under either P deficient or sufficient conditions (**Supplementary Figure S6**). The results shown in **Figure 3D** and **Supplementary Figure S6** were calculated using *OsHistone3* as the reference gene. Similar results were obtained when *OsACTIN* was used as the reference gene (data not shown).

### Suppression of *OsWRKY28* Affects Root Development

**FIGURE 4:**
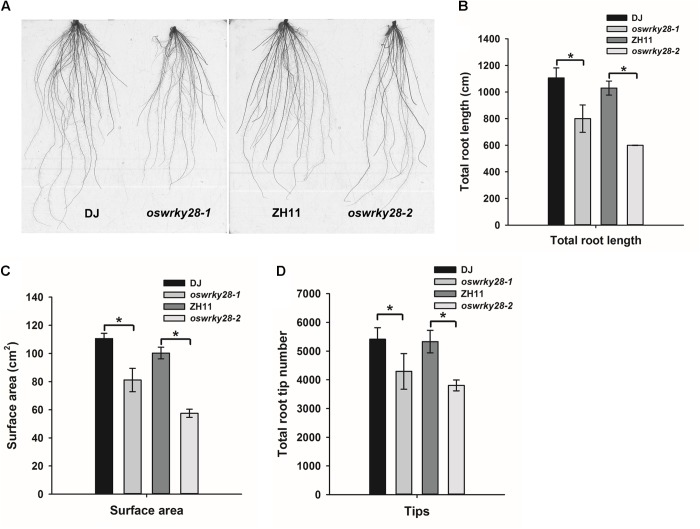
Root architecture of wild-type and *oswrky28* plants. Two wild-types and *oswrky28* T-DNA mutants were grown under 100 μM Pi conditions for 3 weeks. **(A)** Root architectural details. **(B)** Total root length. **(C)** Root surface area. **(D)** Total root tip number. Asterisks indicate statistically different at *P* < 0.05 (Tukey’s test). Values are means ± SE (*n* = 4 per genotype).

Under normal hydroponic conditions, the two mutants showed altered root system architecture (RSA) compared with WTs (**Figure 4A**). Total root length, root surface area and the number of root tips (including the tips of both primary and lateral roots) of the mutants were all significantly (*P* < 0.05) smaller, by 20 – 30%, than those of their respective WT (**Figures 4B–D**). The difference was more pronounced between *oswrky28-2* and ZH11 than that between *oswrky28-1* and DJ. The results suggest that *OsWRKY28* plays an important role in root development, especially the development of lateral roots.

### Suppression of *OsWRKY28* Affects Grain Yield

**FIGURE 5:**
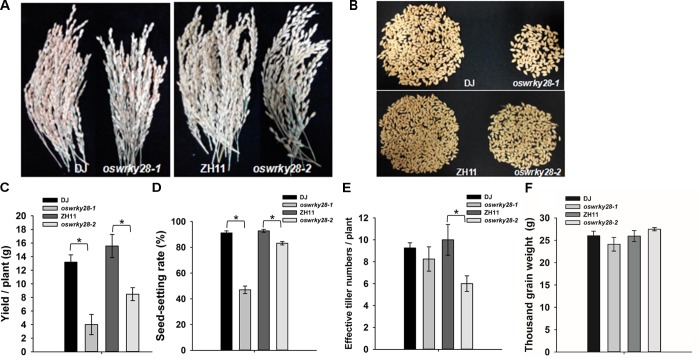
Agronomic trait comparison of the wild-type and *oswrky28* mutants in the soil pot experiment. **(A)** Comparison of the spikelets of wild-type and *oswrky28* plants. **(B,C)** Seed yield per plant of wild-type and *oswrky28*. **(D)** Seed-setting rate; **(E)** Effective tiller number; **(F)** Thousand grain weight. Asterisks indicate significantly different at *P* < 0.05 (Tukey’s test, *n* = 4).

Mutant and WT plants were grown to maturity in a soil pot experiment. The grain yields of *oswrky28-1* and *oswrky28-2* mutants were found decreased significantly, by 69 and 45%, respectively, compared with WTs (**Figures 5A–C**). The average seed-setting rates of *oswrky28* mutants were also significantly lower than their WTs, by 48 and 10% in *oswrky28-1* and *oswrky28-2*, respectively (**Figure 5D**). The average effective tiller number of *oswrky28-2* was 40% lower than WT (ZH11), whereas there was no significant difference between *oswrky28-1* and DJ (**Figure 5E**). In contrast, the 1000 grain weight was not altered in the mutants (**Figure 5F**). In addition, there were no significant differences in As or total P concentrations in straws and grain between *oswrky28* mutants and WTs (**Supplementary Figure S8**).

### Transcriptomic Comparison of *Oswrky28* Mutant and Wild-Type

To explore the possible mechanisms for the effects of *oswrky28* mutants on As and P accumulation and plant development, we analyzed the root and shoot transcriptomes of *oswrky28-1* and WT (DJ) by RNA-seq. The 4-week old plants were grown under normal hydroponic conditions, and the mutant showed different root architecture compared to WT as shown in **Figure 4A**. Compared with WT, 219 and 128 genes were significantly (fold change > 2) up-regulated in mutant roots and shoots, respectively, whilst 54 and 256 genes were significantly down-regulated (**Supplementary Data Sheet S1**).

**FIGURE 6:**
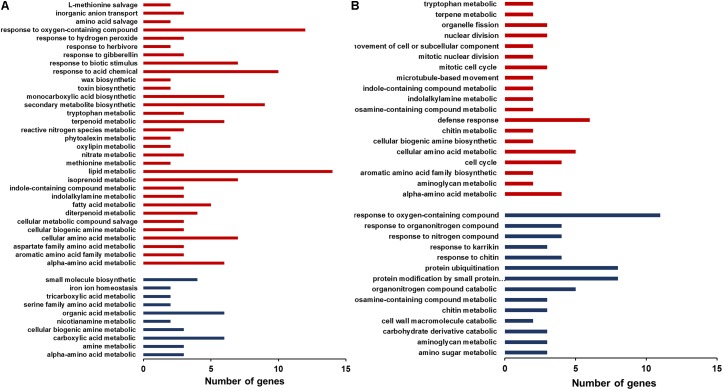
Gene ontology (GO) enrichment analysis of differently expressed genes in roots **(A)** and shoots **(B)** of WT and *oswrky28-1* mutant. GO categories are clustered by the biological processes. Red and blue colors represent the genes up- and down-regulated, respectively.

The differentially expressed genes were classified by GO (Gene ontology) enrichment analysis. GO categories are clustered by the biological process. In the root GO enrichment results, the genes that are involved in the responses to oxygen-containing compounds and in lipid metabolism were more numerous than other categories (**Figure 6A**). In addition, the genes responsive to biotic stimulus, herbivore and gibberellin also exhibited increased expression in the mutant. In contrast, some genes functioning in small molecule biosynthesis, organic acid metabolism and carboxylic acid metabolism were down-regulated in the mutant roots. In the shoots, genes participating in the defense responses, cellular amino acid metabolism, cell cycle and alpha-amino acid metabolic process were up-regulated, whereas down-regulated genes included those involved in the responses to oxygen-containing compounds, protein ubiquitination and protein modification by small protein conjugation (**Figure 6B**). The increased expression of genes responsive to biotic stimulus or involved in defense responses in the mutant is consistent with the study of Delteil et al. (2012), which also showed elevated expression of pathogenesis related genes in T-DNA insertion *oswrky28* mutants (Delteil et al., 2012).

**FIGURE 7:**
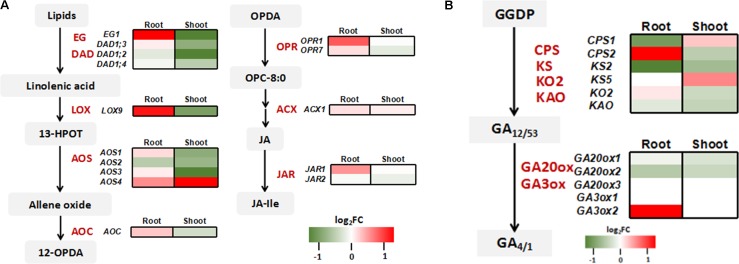
Expression profiling of genes involved in jasmonic acid (JA) and gibberellin (GA) synthesis. **(A)** Heat map comparison of genes involved in JA biosynthesis between *oswrky28* mutant and WT. The main enzymes included extra glume 1 (EG1), defective in anther dehiscence1 (DAD1), 13-lipoxygenases (LOX), allene oxide synthase (AOS), allene oxide cyclase (AOC), OPDA reductase (OPR), acyl-CoA oxidase (ACX) and GH3 enzyme jasmonate resistant (JAR) (Wasternack and Hause, 2013). **(B)** Heat map comparison of genes involved in GA biosynthesis between *oswrky28* mutant and WT. The main enzymes included Ent-copalyl diphosphate synthase (CPS), ent-kaurene synthase (KS) ent-kaurene oxidase 2 (KO2), gibberellin 20-oxidase gene (GA20ox) and gibberellins 3β-hydroxylase gene (GA3ox). The genes involved in GA synthesis process from trans-geranylgeranyl diphosphate (GGDP) were analyzed (Ayano et al., 2014). Color scale represents log_2_ (fold change in gene expression between mutant and WT).

Consistent with the quantitative RT-PCR results (**Figure 3D**), the expressions of Pi transporter genes and As related genes were not affected by *OsWRK28* mutation in the RNA-seq analysis (**Supplementary Figure S9**). Because not only As(V)/Pi uptake but also plant development was affected in the mutants, we checked the phytohormone related genes in the RNA-seq data. Three gibberellic acid (GA) response genes (Os02g0106100, *OsINV3*, Os06g0569500, *OsKO4* and Os06g0728700) and two lipoxygenases (LOX) genes (Os02g0194700, *OsLOX1* and Os12g0559200, *OsLOX11*) that are involved in JA synthesis were enriched in the up-regulated genes in the mutant roots. GA and JA are important phytohormones modulating growth and development throughout the plant life cycle (Olszewski et al., 2002; Liu et al., 2015). We further analyzed the expression profiles of genes involved in the biosynthesis of GA and JA. No consistent changes were found in the GA biosynthesis genes in either roots or shoots (**Figure 7B**). However, JA biosynthesis genes were markedly up-regulated in the roots but down-regulated in the shoots of the mutant compared with WT (**Figure 7A**). The gene encoding GH3 enzyme JASMONATE RESISTANT (JAR) for the conjugation of JA with isoleucine to activate the phytohormone was also up-regulated in the mutant roots.

### Exogenous JA Treatment

**FIGURE 8:**
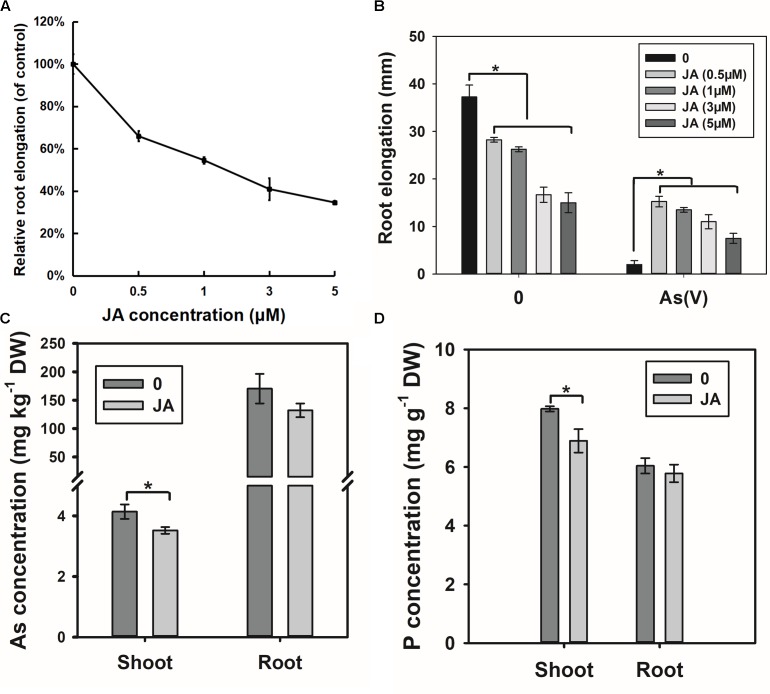
Effect of exogenous JA treatment on root growth and arsenate uptake. **(A)** Root elongation response to the JA treatment. Root elongation was measured after 48 h of JA treatment. Relative root elongation was calculated to the control. Data are means ± SE (*n* = 10). **(B)** Arsenate tolerance of plants after JA treatment. 48 h-root elongation was measured to assess arsenate tolerance. The concentration of arsenate was 2 μM. Data are means ± SE (*n* = 10). **(C)** Effect of JA treatment on As concentrations in roots and shoots. **(D)** Effect of JA treatment on *P* concentrations in roots and shoots. Three-week old plants were pretreated by 0.5 μM JA for 2 days, and then treated by 5 μM arsenate in 1/2 kimura solution for 3 days. Data are means ± SE (*n* = 3). Asterisks indicate significantly different at *P* < 0.05 (Tukey’s test).

The RNA-seq results suggest that OsWRKY28 may affect JA biosynthesis, which could in turn influence As(V) uptake and root development. To test this hypothesis, we treated the WT rice seedlings with exogenous JA. Exogenous JA inhibited root elongation in a dose-dependent manner (**Figure 8A**). Interestingly, JA pre-treated plants showed significantly less inhibition of root elongation by 2 μM As(V) than non-treated plants (**Figure 8B**), suggesting that JA pretreatment alleviates As(V) toxicity. Furthermore, JA pretreatment decreased the As concentrations in the roots and shoots, with the effect being significantly for shoot As concentration (**Figure 8C**). P concentration in the shoots was also significantly decreased by JA pre-treatment (**Figure 8D**). The phenotypes of JA pretreatment, including decreased root development and lower As(V)/P uptake and/or translocation to the shoots, were similar to those of *oswrky28* mutants.

## Discussion

*OsWRKY28* has been reported to be involved in the innate immune responses to the infection of rice blast fungus (Peng et al., 2010; Delteil et al., 2012; Chujo et al., 2013). Whether *OsWRKY28* is involved in other physiological processes has not been characterized. In the present study, we found that OsWRKY28 is localized in the nucleus (**Figures 2A–D**), which is consistent with it being a transcription factor. Our results show that the gene is expressed in different tissues of rice plants, including the reproductive organs such as stamen, stigma and ovary. Strong expression of *OsWRKY28* was found in the lateral roots and the tips of primary roots (**Figures 2E–J**). These patterns of tissue expression are consistent with the phenotypes of reduced root growth and seed setting in the mutants (**Figures 4, 5**).

In this study, we found that the expression of *OsWRKY28* was induced markedly by As(V) (**Figure 1**). The response to As(V) exposure was very rapid, although the initial burst in the induced expression was followed by a gradual decline. In the *oswrky28* mutants, As and Pi concentrations in the shoots of mutants were significantly lower than those in WTs in a short-term As(V) exposure experiment (**Figure 3A**), suggesting that the suppression of *OsWRKY28* disturbed the translocation of As(V) and Pi from the roots to the shoots. In Arabidopsis, AtWRKY6 was found to be induced by As(V) and act to down-regulate Pi transporters to reduce As(V) absorption (Castrillo et al., 2013). Although *OsWRKY28* responds to As(V) similarly as *AtWRKY6*, none of the 13 genes encoding Pi transporters tested in our study showed a consistent difference in expression between *oswrky28* mutants and WTs (**Figure 3D**). Because As(V) was readily reduced to As(III) after absorption by rice roots (Shi et al., 2016; Xu et al., 2017), we also investigated the expression of genes related to As(III) uptake and detoxification (Ma et al., 2008; Song et al., 2014; Hayashi et al., 2017; Uraguchi et al., 2017; Yamazaki et al., 2018), but found no consistent differences between mutants and WTs (**Supplementary Figure S7**). It is possible that the lower accumulations of As and P in the mutant shoots may not result from lower expression of Pi/As(V) transporter genes, but may be an indirect result of the metabolic disturbance. Although As and P accumulation in the shoots was reduced in the mutants in short-term hydroponic experiment, no significant differences were found in the As concentrations in the straws and grains between mutants and WTs in the soil pot experiment (**Supplementary Figure S8**). This is not surprising, because the dominant As species in anaerobic paddy soils is As(III) (Khan et al., 2010; Stroud et al., 2011), which is taken up by the Si transporters (Ma et al., 2008). Thus, the Pi/As(V) uptake pathway contributes little to As accumulation in rice grain and straw grown under flooded conditions (Wu et al., 2011).

In addition to the effect on As(V) and Pi translocation, mutation in *OsWRKY28* also has a profound effect on the root architecture, tillering and grain yield. The two *oswrky28* mutants produced fewer root tips, smaller total root length and root surface area, fewer tillers and lower seed setting rates than WTs (**Figure 4**), suggesting that *OsWRKY28* is a positive regulator of plant development. It is unlikely that the altered root architecture and decreased fertility in the mutants were caused by P deficiency, because mutation in *OsWRKY28* did not affect root P concentration significantly and the decrease in shoot P concentration was relatively small (short-term hydroponic experiment) or insignificant (soil pot experiment). It is possible that OsWRKY28 affects plant development by regulating hormone homeostasis or signaling pathway. Whilst *oswrky28* mutants showed the phenotype of retarded root development, overexpression of *OsWRKY28* did not appear to alter root growth significantly (**Supplementary Figure S4**). WRKY genes were reported to play roles in regulating plant hormone synthesis (Xie et al., 2005; Qiu et al., 2007; Peng et al., 2012). Some WRKY genes are known to influence root architecture by affecting plant hormones signaling or genes expression which are important for root development in rice. For example, Zhang et al. (2008) showed that overexpression of *OsWRKY31* resulted in fewer and shorter lateral roots compared to WT plants, possibly by disturbing the auxin response or transport (Zhang et al., 2008). In the present study, RNA-seq data showed that GA and JA related genes were enriched in GO analysis and genes in the JA biosynthesis pathway were up-regulated markedly in mutant roots. Exogenous methyl jasmonate (MeJA) was reported to reduce As uptake and oxidative stress in *Brassica napus* (Farooq et al., 2016). Similarly, we found that exogenous JA inhibited root growth and decreased As(V) uptake. These phenotypes were similar to those of *oswrky28* mutants, suggesting that mutations in *OsWRKY28* may alter the endogenous JA level and thus affect root growth and As(V) uptake. In contrast, *oswrky28* mutant had decreased expression of JA biosynthesis genes in the shoots. JA is important for reproductive development in rice. JA biosynthesis mutants of rice showed disordered spikelet development, altered flower closing and anther dehiscence, resulting in a lower fertility (Cai et al., 2014; Xiao et al., 2014; Chen et al., 2016). It is possible that decreased seed setting in the *oswrky28* mutants was caused by a decreased JA level in the flower organs during the reproductive stage.

The expression of *OsWRKY28* was induced not only by As(V), but also by As(III), Cd, Cu and H_2_O_2_. It is known that these treatments can cause oxidative stresses to plants. A number of studies have shown that JA can alleviate oxidative stresses caused by heavy metal exposure. For example, applications of exogenous methyl jasmonate (MeJA) alleviated the oxidative damages in *Kandelia obovata* and *Capsicum frutescens* induced by Cd stress (Yan et al., 2013; Chen et al., 2014). Exogenous JA increased the accumulation of chlorophyll and carotenoid in *Cajanus cajan* and neutralized the toxic effect of Cu (Poonam et al., 2013). The alleviation of As(V) toxicity by exogenous JA was also observed in our experiment (**Figure 8B**). These observations suggest that induced *OsWRKY28* expression by As, Cu, Cd, and H_2_O_2_ could affect JA homeostasis in response to oxidative stresses.

In summary, the expression of *OsWRKY28* responded to oxidative stresses induced by As(V) and other heavy metals or H_2_O_2_. Mutations in *OsWRKY28* resulted in decreased translocation of As from the roots to the shoots, but this effect was not related to changes in the expression of Pi/As(V) transporter genes. Mutations in *OsWRKY28* affected the development of roots, tillers and reproductive organs in rice. It is possible that the observed phenotypes in the *oswrky28* mutants may be related to altered homeostasis of phytohormones, especially JA. Further investigations are required to elucidate the regulatory mechanisms of *OsWRKY28*.

## Author Contributions

F-JZ and PW designed the research work. PW, XX, ZT, and WZ performed the experiments. PW and X-YH analyzed the data. PW and F-JZ wrote the manuscript.

## Conflict of Interest Statement

The authors declare that the research was conducted in the absence of any commercial or financial relationships that could be construed as a potential conflict of interest.

## Supplementary Material

FIGURE S1 The expression of *OsWRKY28* responsive to oxidative stress. The transcript levels of *OsWRKY28* exposed to arsenate and other oxidative stresses. Three-week-old plants were treated with 1.5 μM As^5+^, 20 μM As^3+^, 3 μM Cd, 2 μM Cu, 500 μM H_2_O_2_ without Pi supply for 12 h. The relative expression of OsWRKY28 were normalized to the expression level of control. *Histone3* was used as the reference gene. Data are means ± SE (*n* = 3 biological replicates).

FIGURE S2 OsWRKY28 expression pattern. OsWRKY28 expression pattern throughout entire growth. The data was obtained from RiceXPro database (http://ricexpro.dna.affrc.go.jp/category-select.php).

FIGURE S3 Characterization of the *oswrky28* mutants. **(A)** Diagram of T-DNA insertion mutants of the *OsWRKY28* gene. **(B)** Confirmation of the T-DNA insertion position by PCR analysis. **(C)** The expression of *OsWRKY28* in wild-type and mutants.

FIGURE S4 Element concentrations in shoots **(A)** and roots **(B)** of WT and *oswrky28* mutants. Plants were hydroponically cultivated in 1/2 kimura for 3 weeks. Data are means ± SE (*n* = 4). Asterisks represent means are significantly different at *P* < 0.05 (Tukey’s test).

FIGURE S5 Phenotypes of *OsWRKY28* over-expression lines in hydroponic experiments. Photo of wild-type and over-expression plants **(A)**. The expression level of *OsWRKY28* in overexpression lines **(B)**. Dry weight of wild-type and overexpression (OX) lines hydroponically grown for 4 weeks **(C,D)**. Arsenate **(E,F)** and phosphate **(G,H)** concentrations in shoots and roots of wild-type and overexpression lines.

FIGURE S6 The expression levels of different phosphate transporter genes in the roots of WT and *wrky28*. Data are means ± SE (*n* = 3). Two-week-old plants were separated to two phosphate levels, 0 μM Pi and 91 μM Pi, for 1 week. The gene expressions of *IPS1, PT1, PT2, PT6* and *PT8* in shoots **(A–E)** and roots **(F–J)** were quantified. The gene relative expression of WTs and mutants were normalized to the expression level of DJ cultivated with 91 μM phosphate. *Histone3* was used as the reference gene. Data are means ± SE (*n* = 3 biological replicates).

FIGURE S7 The expression levels of genes in the roots of WTs and *wrky28* mutants. Data are means ± SE (*n* = 3). Three-week-old plants were used to test the expressions of *Lsi1, Lsi2, PCS1, PCS2, ABCC1* and *PHF1* in roots. The gene relative expression of mutants were normalized to their wild-types. *Histone3* was used as the reference gene. Data are means ± SE (*n* = 3 biological replicates).

FIGURE S8 Concentrations of Arsenate **(A,B)** and phosphate **(C,D)** in the straws **(A,C)** and grains **(B,D)** of WT and *oswrky28* mutants in soil pot experiments.

FIGURE S9 Heat map analysis of phosphate transporter and arsenic related genes in RNA-seq. Gene expression level comparison of genes in shoots **(A)** and roots **(B)**. Colors represent the expression profile of every gene in mutant and WT. The number is the FPKM (Fragments Per Kilobase of exon model per Million mapped reads) value.

TABLE S1 Primers used in this study.

DATA SHEET S1 Differentially expressed genes in roots and shoots between wild-type and *oswrky28* mutant.
