# Supplementary material for: OsWRKY28 Regulates Phosphate and Arsenate Accumulation, Root System Architecture and Fertility in Rice
Source: Front Plant Sci. 2018 Sep 12;9:1330. doi: 10.3389/fpls.2018.01330 (PMC6143681; doi:10.3389/fpls.2018.01330)
Supplement: FIGURE S1 — The expression of OsWRKY28 responsive to oxidative stress. The transcript levels of OsWRKY28 exposed to arsenate and other oxidative stresses. Three-week-old plants were treated with 1.5 μM As5+, 20 μM As3+, 3 μM Cd, 2 μM Cu, 500 μM H2O2 without Pi supply for 12 h. The relative expression of OsWRKY28 were normalized to the expression level of control. Histone3 was used as the reference gene. Data are means ± SE (n = 3 biological replicates). [file Presentation_1.PDF]

OsWRKY28 regulates phosphate and arsenate accumulation, root system architecture and fertility in rice

Peitong Wang, Xuan Xu, Zhong Tang, Wenwen Zhang, Xin-Yuan Huang, Fang-Jie Zhao*

State Key Laboratory of Crop Genetics and Germplasm Enhancement, College of Resources and Environmental Sciences, Nanjing Agricultural University, Nanjing 210095, China

SUPPLEMENTARY MATERIALS

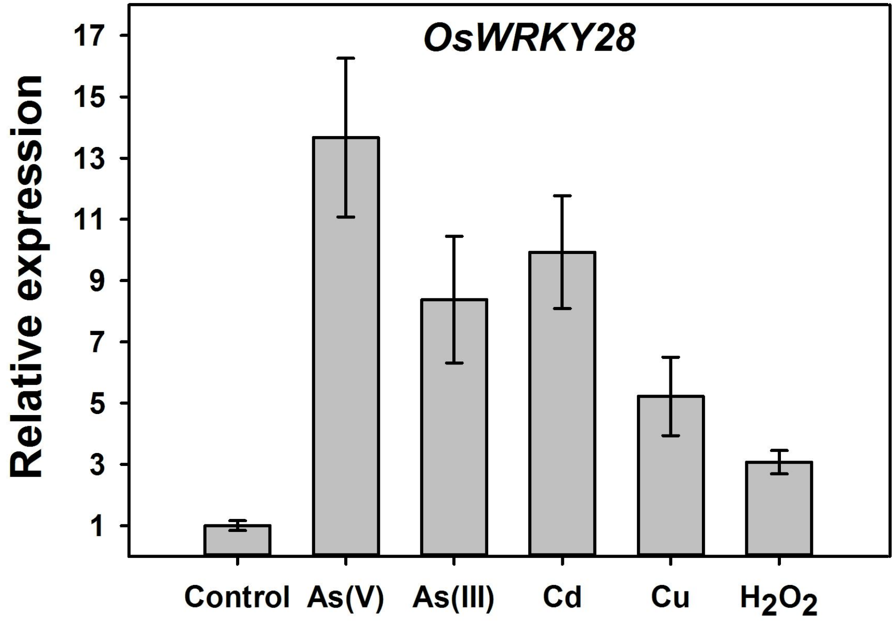

Fig. S1. The expression of *OsWRKY28* responsive to oxidative stress. (A) The transcript levels of *OsWRKY28* exposed to arsenate and other oxidative stresses. Three-week-old plants were treated with 1.5 μM As^{5+}, 20 μM As^{3+}, 3 μM Cd, 2 μM Cu, 500 μM H_2O_2 without Pi supply for 12 h. The relative expression of *OsWRKY28* were normalized to the expression level of control. *Histone3* was used as the reference gene. Data are means ± SE (*n* = 3 biological replicates).

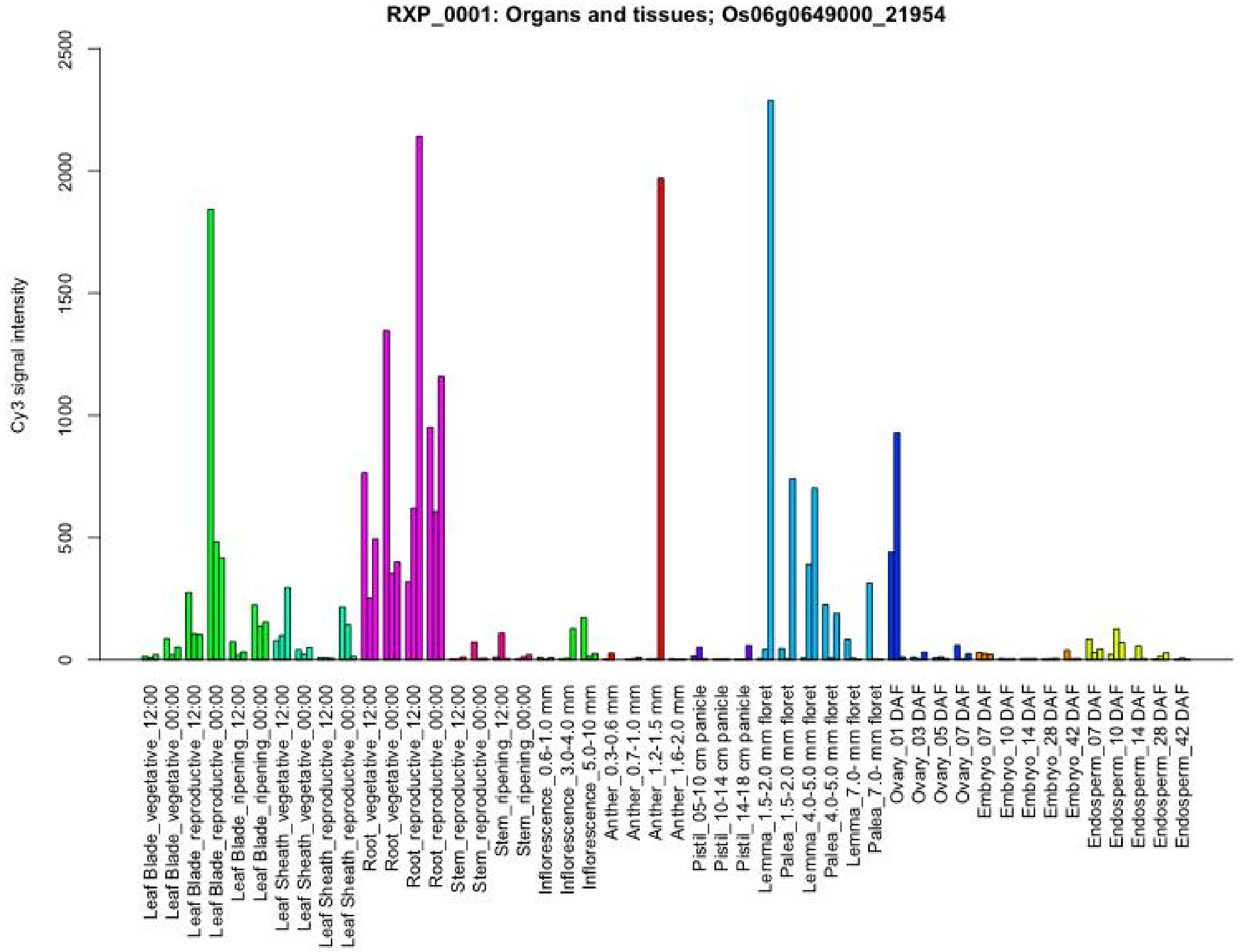

Fig. S2. *OsWRKY28* expression pattern. *OsWRKY28* expression pattern throughout entire growth. The data was obtained from RiceXPro database (http://ricexpro.dna.affrc.go.jp/category-select.php).

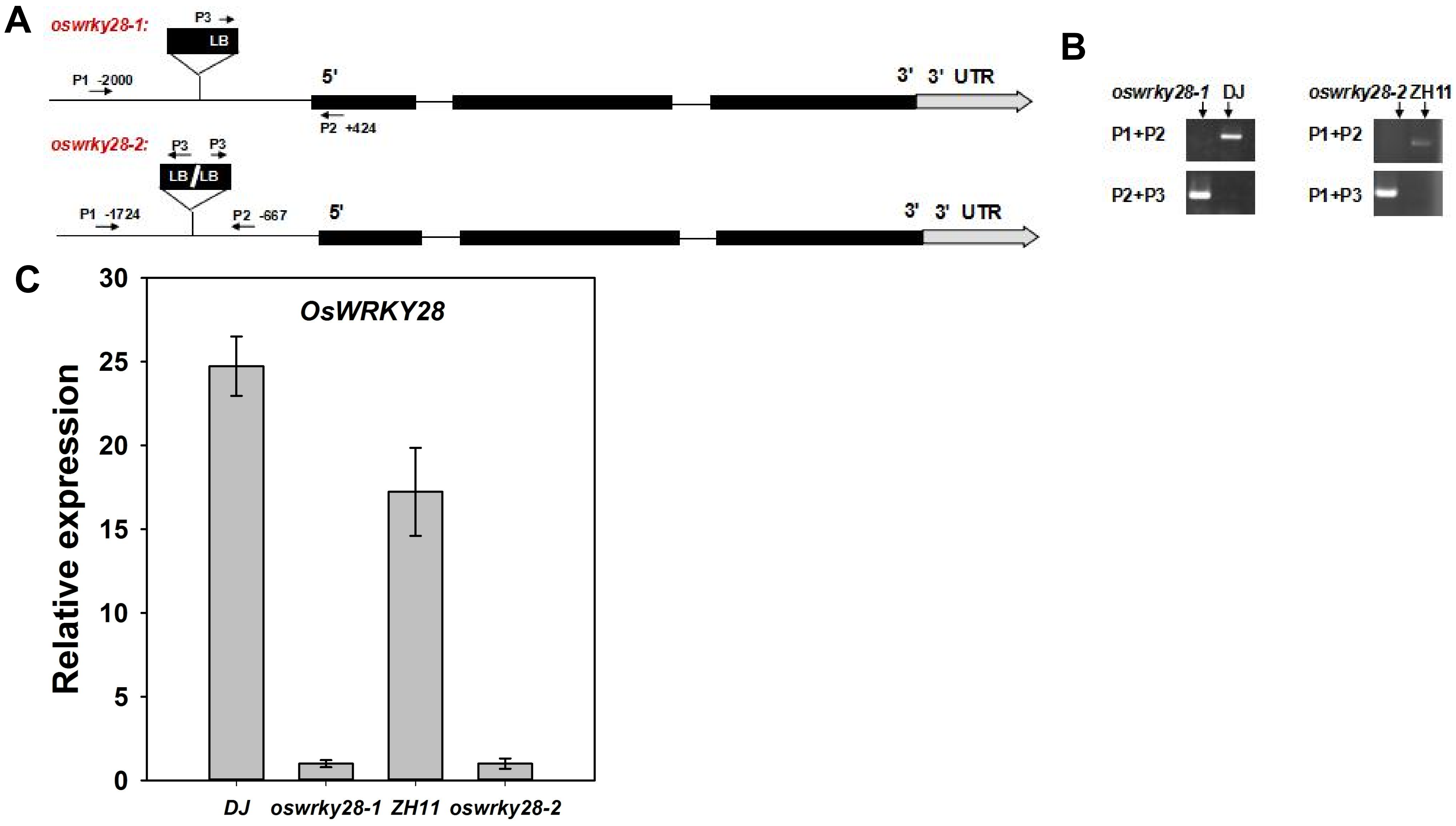

Fig. S3. Characterization of the os*wrky28* mutants. (A) Diagram of T-DNA insertion mutants of the *OsWRKY28* gene. (B) Confirmation of the T-DNA insertion position by PCR analysis. (C) The expression of *WRKY28* in wild-type and mutants. Data are means±SE (*n*=3).

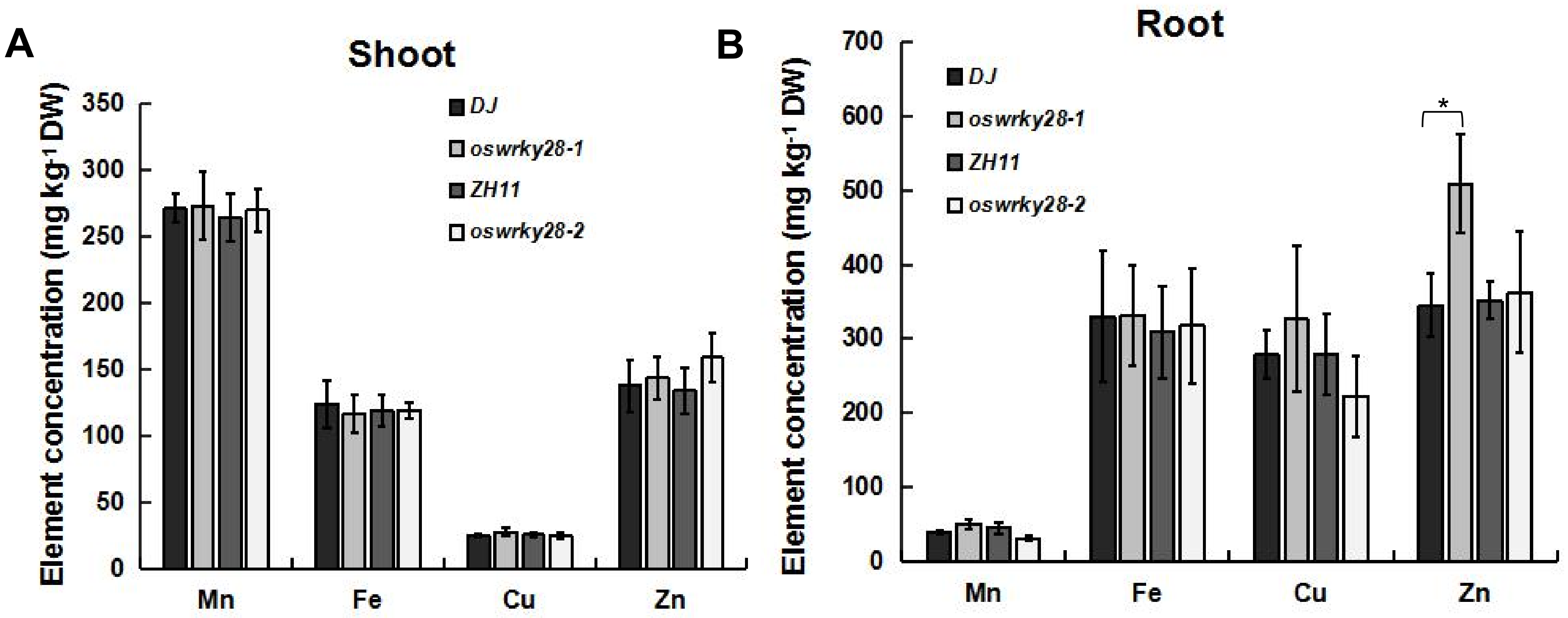

Fig. S4. Element concentrations in shoots (A) and roots (B) of WT and *wrky28* mutants. Plants were hydroponically cultivated in 1/2 kimura for 3 weeks. Data are means ± SE (n = 4). Asterisks represent means are significantly different at $P < 0.05$ (Tukey's test).

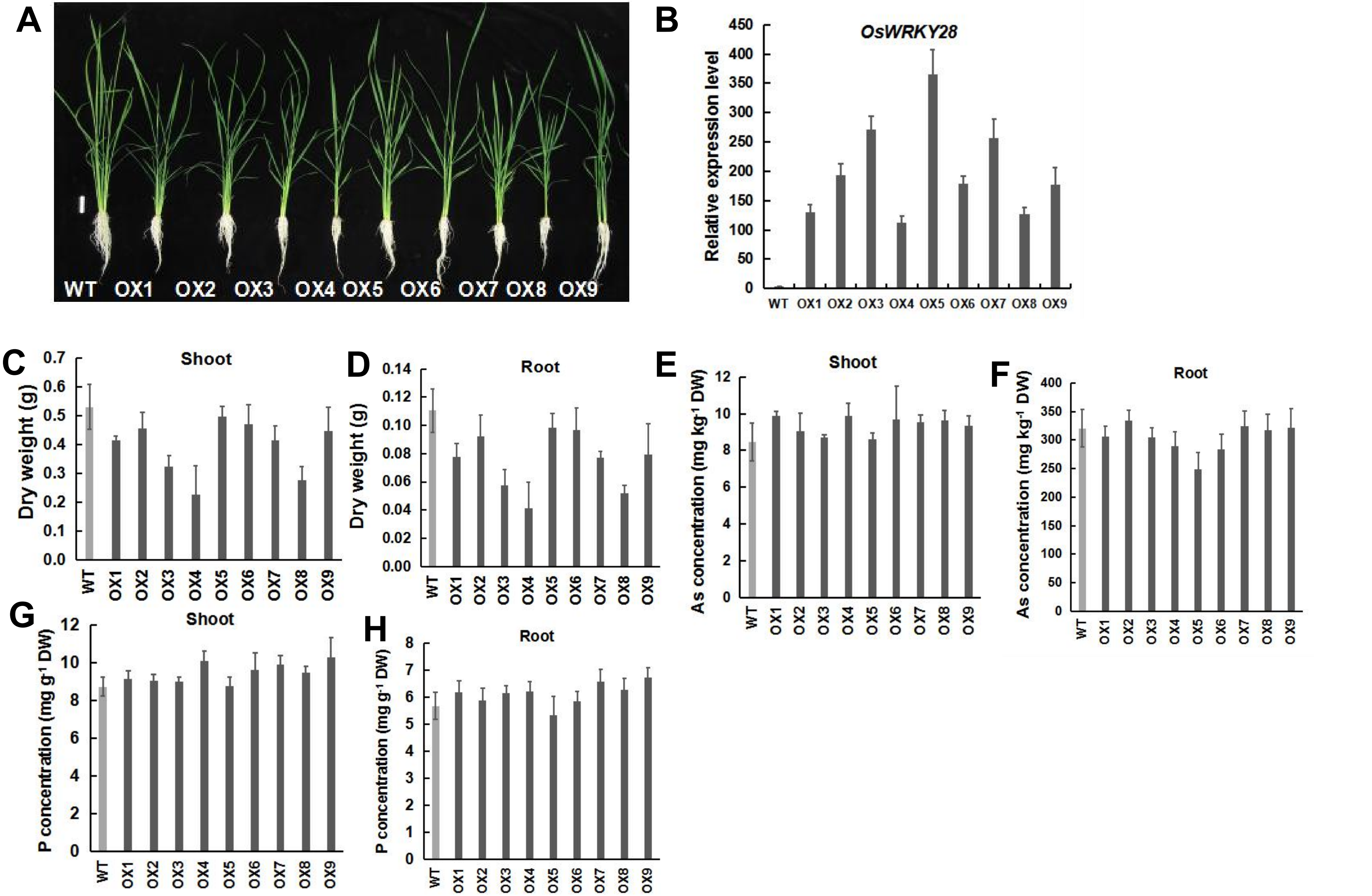

Fig. S5. Phenotypes of *WRKY28* over-expression lines in hydroponic experiments. The expression level of *WRKY28* in overexpression lines (B). Dry weight of wild-type and overexpression(OX) lines hydroponically grown for 4 weeks (C, D). Arsenate (E, F) and phosphate (G, H) concentrations in shoots and roots of wild-type and overexpression lines. Data are means ± SE (*n* = 3).

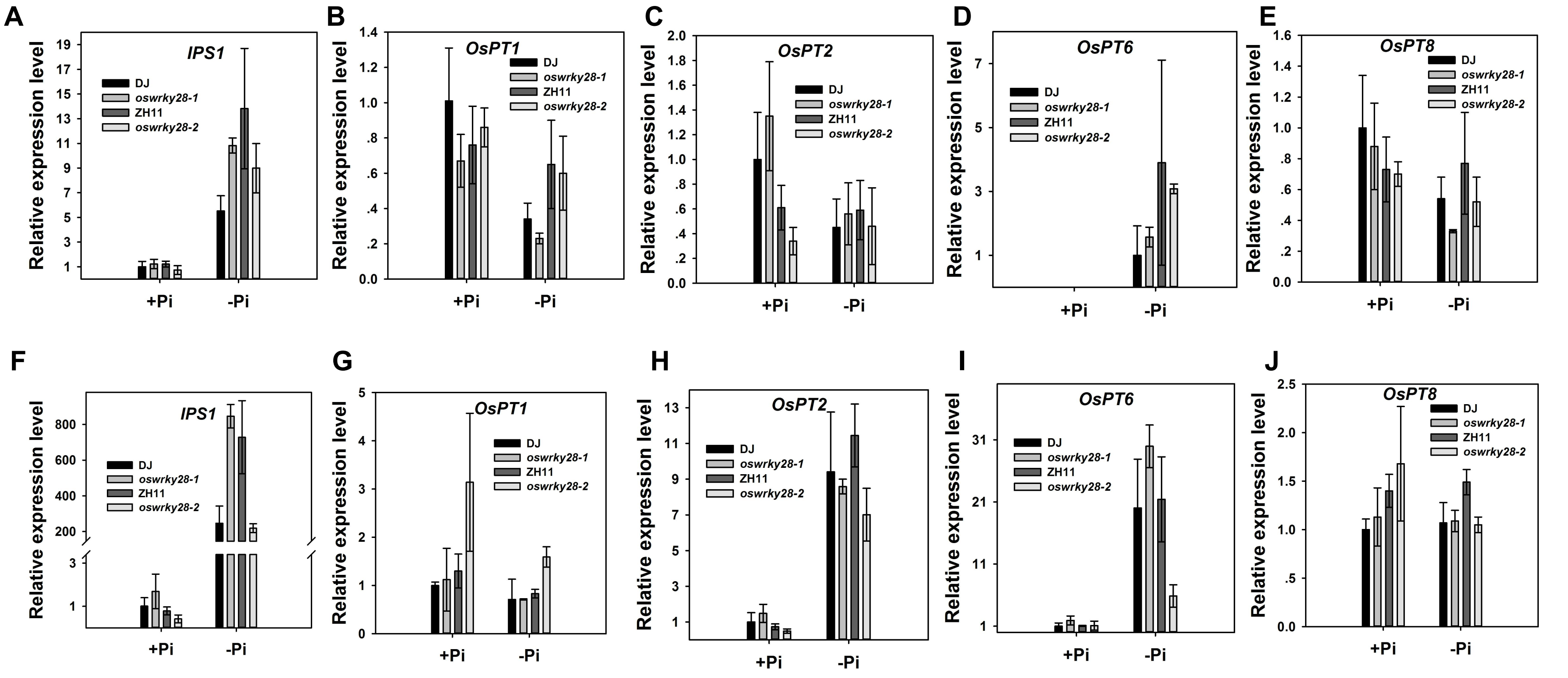

Fig. S6. The expression levels of different phosphate transporter genes in the roots of WT and *wrky28* without arsenate treatment. Data are means ± SE (*n* = 3). Two-week-old plants were separated to two phosphate level, 0μM Pi and 91μM Pi, for 1 week. The gene expressions of *IPS1*, *PT1*, *PT2*, *PT6* and *PT8* in shoots (A-E) and roots (F-J) were tested. The gene relative expression of WTs and mutants were normalized to the expression level of DJ cultivated with 91μM phosphate. *Histone3* was used as the reference gene. Data are means±SE (*n* = 3 biological replicates).

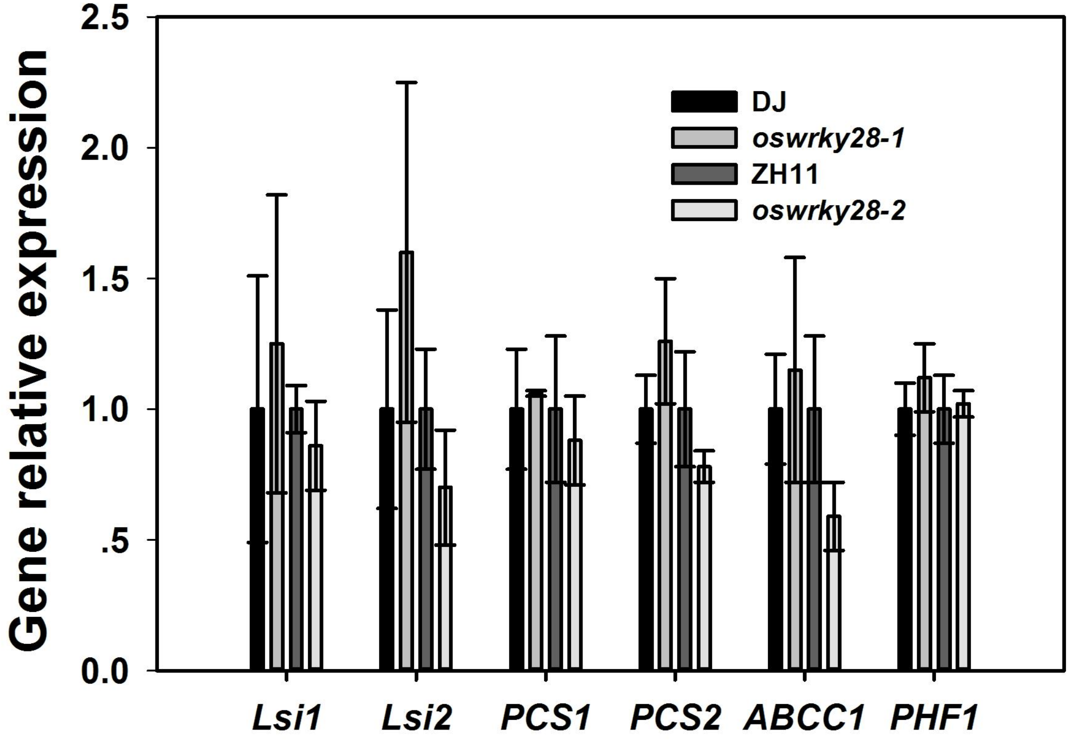

Fig. S7. The expression levels of genes in the roots of WTs and *wrky28 mutants*. Data are means ± SE (*n* = 3). Three-week-old plants were used to test the expressions of *Lsi1*, *Lsi2*, *PCS1*, *PCS2, ABCC1* and PHF1 in roots. The gene relative expression of mutants were normalized to their wild types. *Histone3* was used as the reference gene. Data are means±SE (*n* = 3 biological replicates).

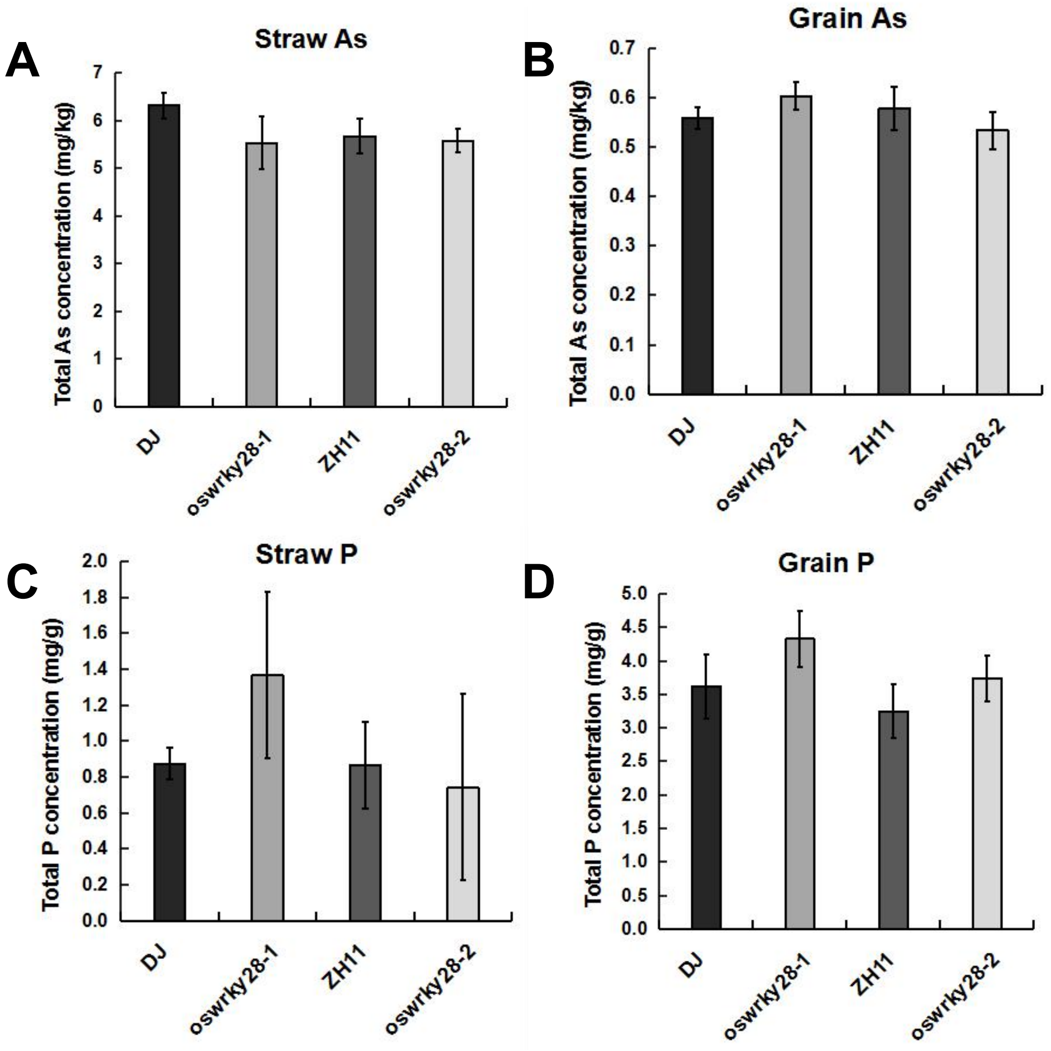

Fig. S8. Concentrations of Arsenate(A, B) and phosphate(C, D) in the straws (A, C) and grains (B, D) of WT and *wrky28* mutants in soil pot experiments. Data are means ± SE (n = 4).

A

	PT1	*PT2*	*PT3*	*PT4*	*PT5*	*PT6*	*PT7*	*PT8*	*PT9*	*PT10*	*PT11*	*PT12*	*PT13*	*PHO1;2*	*PHF1*	*Lsi1*	*Lsi2*	*PCS1*	*ABCC1*
**WT**	379	0	0	1	0	0	0	83	0	0	0	0	0	2	67	4	0	3	260
wrky28	246	0	0	1	0	0	0	56	0	0	0	0	0	3	60	3	0	2	276

B

	PT1	*PT2*	*PT3*	*PT4*	*PT5*	*PT6*	*PT7*	*PT8*	*PT9*	*PT10*	*PT11*	*PT12*	*PT13*	*PHO1;2*	*PHF1*	*Lsi1*	*Lsi2*	*PCS1*	*ABCC1*
**WT**	371	1	0	8	0	0	0	56	0	0	0	0	0	49	78	1649	869	1	222
wrky28	369	2	0	7	0	1	0	65	0	0	0	0	0	47	73	1908	1042	1	217

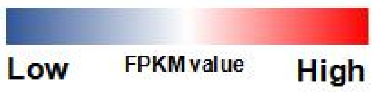

Fig. S9. Heat map analysis of phosphate transporter and arsenic related genes in RNA-seq. Gene expression level comparison of genes in shoots(A) and roots (B). Colors represent the expression profile of every gene in mutant and WT. The number is the FPKM (Fragments Per Kilobase of exon model per Million mapped reads) value.

Table S1. Primers used in this study

	Primer sequence (5'-3')
Oswrky28-1 T-DNA LP	ACACTAACTACTAGGACTACTGGCA
Oswrky28-1 T-DNA RP	TCCAGCGCCTCAACCTGG
Oswrky28-1 T-DNA BP	ATGGCAGTGAATTAACATAGC
Oswrky28-2 T-DNA LP	AGTACAATGCATCTTCCCCG
Oswrky28-2 T-DNA RP	TTGCTATATTTTTCTTCAAAATTCATC
Oswrky28-2 T-DNA BP	GGAAGGGTCTTGCGAAGT
GUS-OsWRKY28	CTTATGCATGCGGCCGCTTAATATTAATTCGACCATGCATCTCAT
	GAAACAAGGCGGACCTTTGCACGGCTGGATCGATGGACACAAGAT
Over-OsWRKY28	CGGGGTACCATGGCTAAGATGCTTCCTCCT
	GGACTAGTTCAGTTCTTGGTCGGCGAGA
YFP-OsWRKY28	GGGGACAAGTTTGTACAAAAAAGCAGGCTTCATGGCTAAGATGCTTCCTC
	GGGGACCACTTTGTACAAGAAAGCTGGGTTCAGTTCTTGGTCGGCGAGAG
Q-PCR-Histone3	GGTCAACTTGTTGATTCCCCTCT
	AACCGCAAAATCCAAAGAACG
Q-PCR-ACTIN	CAACACCCCTGCTATGTACG
	CATCACCAGAGTCCAACACAA
Q-PCR-OsWRKY28	GAAGGATGGGTATCAATGGC
	GTTGTGCTCGCCCTCGTA
Q-PCR-OsIPS1	TTGGCAATTATTCGGTGGAT
	ACCATTTCACCATCCTCTTTATG
Q-PCR-Lsi1	CGGTGGATGTGATCGGAACCA
	CGTCGAACTTGTTGCTCGCCA
Q-PCR-Lsi1	ATCTGGGACTTCATGGCCC
	ACGTTTGATGCGAGGTTGG
Q-PCR-PCS1	GATCTTCCTGATCCTCTTAAACTG
	GTGGCCAAGTATCTTCATATGC
Q-PCR-PCS2	AGCACATGGGTGGGCATTAAAGAC
	CTCCATATTCCTCCTCTTTACAG
Q-PCR-ABCC1	AACAGTGGCTTATGTTCCTCAAG
	AACTCCTCTTTCTCCAATCTCTG
